# Using Molecular Targets to Predict and Treat Aortic Aneurysms

**DOI:** 10.31083/j.rcm2309307

**Published:** 2022-09-13

**Authors:** Xiaonan Zhou, Gang Liu, Hao Lai, Chunsheng Wang, Jun Li, Kai Zhu

**Affiliations:** ^1^Department of Cardiac Surgery, Zhongshan Hospital, Fudan University, 200032 Shanghai, China; ^2^Shanghai Institute of Cardiovascular Diseases, Zhongshan Hospital, Fudan University, 200032 Shanghai, China

**Keywords:** aortic aneurysm, molecular target, vascular imaging, diagnosis, risk stratification, treatment

## Abstract

Aortic aneurysms are life-threatening vascular diseases associated with high morbidity, and usually require prophylactic surgical intervention. Current preventative management of aortic aneurysms relies on the diameter and other anatomic parameters of the aorta, but these have been demonstrated to be insufficient predictive factors of disease progression and potential complications. Studies on pathophysiology of aortic aneurysms could fill this need, which already indicated the significance of specific molecules in aortic aneurysms. These molecules provide more accurate prediction, and they also serve as therapeutic targets, some of which are in preclinical stage. In this review, we summarized the inadequacies and achievements of current clinical prediction standards, discussed the molecular targets in prediction and treatment, and especially emphasized the molecules that have shown potentials in early diagnosis, accurate risk assessment and target treatment of aortic aneurysm at early stage.

## 1. Introduction

Aortic aneurysms (AAs) are defined as dilation more than 1.5 times the diameter of normal aortic vessels at the same aortic segment. Among arterial diseases, incidence of AAs is the second highest after atherosclerosis, and AAs are characterized by a high mortality rate and lethal complication. AAs can be classified into thoracic aortic aneurysms (TAAs) (above the diaphragm) and abdominal aortic aneurysms (AAAs) (below the diaphragm) by anatomical locations. Pathophysiological studies have shown that there are differences between the two aneurysms but they also common in various aspects of pathogenesis. Patients with TAAs or AAAs are usually asymptomatic, and most patients are diagnosed accidentally while they are receiving medical attention for other reasons. The prevalence of TAAs is lower than that of AAAs.

One of the largest epidemiological studies in recent years estimated the incidence of TAAs at 7.6 per 100,000 people, an increase from 3.5 to 7.6 per 100,000 over a 12-year period (which might be related to the aging of the population and the advancement of imaging technology) [1]. Of note, the morbidity of males is higher than that of females, but females have worse outcomes. The fatal complications of TAAs are aortic dissection (AD) and aneurysm rupture. The mortality from acute AD rises rapidly over the first 24 hours, increasing 1%–2% per hour; mortality is nearly 50% in the first week, and 90% of all patients die within one year [2]. Moreover, in patients who received emergent treatment and survived to the hospital, the in-hospital mortality was 24% for type A AD and 11% for type B AD [3]. Epidemiological studies have demonstrated higher morbidity of AAAs, but show similar trends to that of TAAs, which increase from 46 to 73 per 100,000 people in the last decade [4].

The clinical diagnosis of AAs relies on modern imaging technologies. Current guidelines use aortic diameter for risk stratification and threshold for prophylactic surgical intervention, and imaging technologies play a crucial role in diameter measurement, such as computed tomography (CT) and magnetic resonance imaging (MRI) [5, 6]. Although endovascular techniques play a significant role in treatment of AAs in recent years, open surgery remains indispensable in the management of AAs involving ascending aorta and aortic arch [5]. However, there is no effective drugs to reverse the disease progression. Once symptoms develop (chest pain, shortness of breath, stroke), the patient might have an unfavorable outcome. Even though modern technologies are sensitive and convenient, by the time they are diagnosed, AAs may have already progressed considerably after a long period of subclinical pathophysiological development. Therefore, there is a urgent need for early diagnosis and therapeutic decision-making to reduce the extremely poor prognosis [5].

Studies have shown that the underlying pathophysiological mechanism of AAs is extensive remodeling of the extracellular matrix (ECM) accompanied by vascular smooth muscle cell (VSMC) loss and elastin fragmentation of the vessel wall [7]. During pathogenesis and progression of AAs, long-term chronic stimulation causes intimal tearing, which is preceded by cystic medial necrosis or medial degeneration [8]. The pathophysiology and progression of AAs involve many molecules and pathways that provide biomarkers to predict the progression of AAs [9]. Thus, by combining in-depth research on the pathological process and application of imaging technologies, more molecular probes have been developed to target pathological tissues. In this review, we discuss inadequacies, advantages and considerations about predictive imaging of aorta and emphasis on the molecular targets and probes that have already shown potential to improve prediction and risk stratification of AAs and treatment to benefit the patients.

## 2. Current Practice to Determine Treatment of AA

### 2.1 Aortic Diameter

Aortic dilatation is a widely accepted risk factor for AD and rupture. Natural complications are rare in moderate size. According to retrospective studies, annual risk of dissection and rupture is 0.08% at diameters of 45 mm, 0.22% of 50 mm, 0.58% of 55 mm in ascending AA, and there is a substantial increase when the diameter exceeds 60 mm to 6.9% yearly [10]. In descending AA, a substantial increase occurs at diameters over 70 mm [11]. For the purpose of preventing aneurysm expansion beyond the critical point, the guidelines recommend prophylatic surgical treatments at 55 mm and 55 to 60 mm for ascending and descending AA respectively [5, 12]. However, patients with connective tissue disease have a significantly high risk of a poor prognosis. For example, patients with Marfan syndrome need lower surgical threshold; preventive surgery is indicated ≥50 mm, or ≥45 mm when accompanying risk factors, such as AA growth >3 mm/year, massive aortic valvular regurgitation or family history of AD [5, 6]. Meanwhile, the bicuspid aortic valve (BAV) is also considered a risk factor to justify surgical intervention at diameters ≥50 mm [13].

Despite the evidence supporting the relationship between aortic dilatation and unfavorable outcomes, a large proportion of catastrophic acute aortic events occur below the criteria for surgical intervention. Retrospective studies have demonstrated that nearly 70% of patients with type A dissection and 80% of patients with type B dissection have aortic diameters ≥55 mm when AD occurs [14, 15]. However, this reflects the late stage of pathology, in which the aortic wall is already severely damaged [16]. Aortic diameter is an insufficient parameter for risk stratification and predicting catastrophic events, in spite of the diameter could predict dissection and rupture at a demography level.

### 2.2 Aorta Anatomic Parameters: Elongation, Tortuosity, Volume and Calcification

**Fig. 1.**
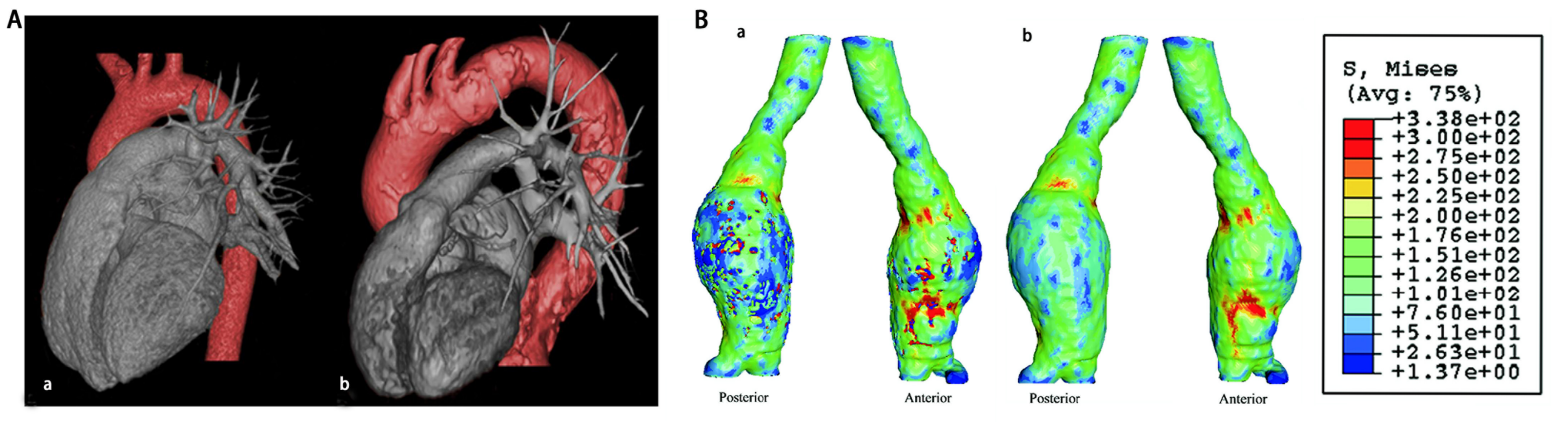
**Aorta anatomic parameters of AA**. (A) 3D modelled CT images of the aorta of a female aged 24 years (a) and a female aged 85 years (b). Reproduced with permission from BMJ Publishing Group Ltd. [17]. (B) 3D wall stress distributions of the two abdominal aortic aneurysm models: (a) unaltered model, (b) no-calcification model. Reproduced with permission from Elsevier [18].

The length of human aorta has been proved increase with age, like aortic diameters (Fig. 1A, Ref. [17, 18]) [17, 19]. Compared with the youth, the length of ascending aorta increases nearly twofold at 80 years of age, which is a great change in length compared with diameter, increasing approximately 12% for length and 3% for diameter per decade [20]. Several lines of evidence suggest that the pressure on the vessel wall increases over ten-fold, as the shape of the ascending aortic changes form straight to curved with normal aortic diameter, blood pressure and cardiac output [21].

Studies have shown a significant increase in volume, while the diameter remains stable in AAs patients [22, 23]. Meanwhile, volume in patients who had surgical treatment are significantly different from that in patients who did not have surgery [24], and this observation could be used in the long-term follow-up of AAs [25]. Although volume measurement can be obtained by modern imaging [26], the value isuncertain and needs further validation.

Calcification is the main characteristic of atherosclerotic cardiovascular disease, and intimal calcification of atherosclerotic plaques strongly correlates with a high risk of cardiovascular events [27]. Fragmentation of intimal calcifications on multi-detector CT is often considered one of the symptoms of unstable AAs (Fig. 1B) [28]. The existing calcification decreases the biomechanical stability of AAs and augments the peak wall shear stress (WSS) of the aorta [18]. For AD, intimal tears are frequently located near or exactly on calcifications in the aorta [29]. However, aortic calcification is not considered as an independent risk factor according to current studies and statistics [30]. Thus, further studies need to be developed on aortic anatomic parameters as predictors and independent risk factors for AAs.

### 2.3 Aortic Hemodynamics

In recent years, hemodynamic assessments have attracted great attention owing to the progress of imaging technology, such as time-resolved three-dimensional phase contrast cardiovascular magnetic resonance (4D-flow CMR). According to the guidelines, CMR and echocardiography are recommended to regularly monitor the aorta [6, 31]. Several studies of 4D-flow CMR have proven the value of this technique as a potential tool to evaluate the WSS of aortic hemodynamics with 3D flow patterns [32, 33]. In addition, certain regions of the aorta with increased WSS accompany the stiffness of the aortic wall and accelerate AAs growth [34]. WSS also influences endothelial cell function and triggers pathways that promote vessel wall remodeling and aortic dilatation [35].

Several studies in patients with BAV have shown that aberrant valve opening can lead to disorganized outflow patterns, which are related to aortic morphology and result in markedly altered regional WSS. In particular, the WSS patterns were significantly different in BAV patients compared with individuals with tricuspid aortic valve (TAV). Outflow patterns vary depending on the types of BAV [36, 37]. TAA formation is one of the frequent complications in BAV patients [38]. Nearly 80% of BAV patients exhibit aortic dilatation with a high risk of AD and rupture [38, 39]. In addition, children and young adults with Marfan syndrome had abnormal WSS, and altered aortic flow patterns are mostly located in the proximal aorta, where the segments are at high risk of aortic dilatation and complications. The two parameters correlated with regional size could be potential markers of risk assessment [40].

## 3. Molecular Targets for the Prediction of AA

### 3.1 Inflammation

In patients with AAs, immunohistochemical analysis and enzyme-linked immunosorbent assay have revealed an inflammatory activity and infiltration in the aneurysm vessel wall [7, 41]. In addition, a large number of CD3+ and CD68+ cells was found in the medial layer of thoracic AA [42]. It has also been demonstrated in positron emission tomography (PET)/computed tomography (CT) that patients who have a progressive course or clinical symptoms of AD exhibiting higher inflammatory cell activity in the aorta than patients who are clinically stable or asymptomatic [43]. Recent data demonstrate that inflammation contributes greatly to aortic wall remodeling, even in the absence of genetic diseases [41, 44]. Basic research and clinical studies have shown that inflammatory cells are associated with medial layer degeneration. These cells are also detected in the wall of the vasa vasorum in the adventitia and at the edges of the ruptured media of dissection [42].

Macrophages are one type of the inflammatory cells that initially infiltrate the aorta. This suggests that macrophages play a major role in ECM degradation processes in the aortic wall of AAs patients regardless of their genetic predisposition [45, 46]. Macrophages and their products, such as collagenases, elastase and cytokines, facilitate inflammatory cell recruitment, increase cytokine stimulation and protease production, and promote neovascularization and lymphocyte differentiation [45]. Recent studies have demonstrated that two types of macrophages, M1 and M2, have distinct functions. M1 macrophages sustaining are accumulate at the site of arterial injury as highly proinflammatory macrophage subse. M2 macrophages found in human AAs samples are mostly associated with inflammation resolution and promote the dissection healing process [47, 48].

Lymphocytes are another inflammatory cell type associated with the inflammatory mechanism of AAs. In lymphocytes, T helper 1 lymphocytes are predominantly related to plaque formation with a proliferative pattern [49]. Meanwhile, T helper 2 lymphocytes, which make up a majority of lymphocytes in aortic lesions, are associated with atherosclerotic development of AAs [50], and secrete interleukins [51]. Recent research suggests that infiltration of T lymphocytes, especially T helper 2 lymphocytes, contribute to modulating the immune response and VSMC apoptosis by activating death-promoting pathways [51].

Recent studies have shown that surface receptor integrin might be a new biomarker in AAs. The integrin CD11b/CD18, also known as macrophage-1 Ag (Mac-1), plays an important role in immune-inflammatory responses as pathogen-associated molecular patterns and damage-associated molecular patterns recognition receptor [52, 53]. Mac-1, which has been reported as a biomarker of inflammatory cells in infarcted myocardium and atherosclerotic plaques [54], predominantly induced cellular immune responses in macrophages [55].

**Fig. 2.**
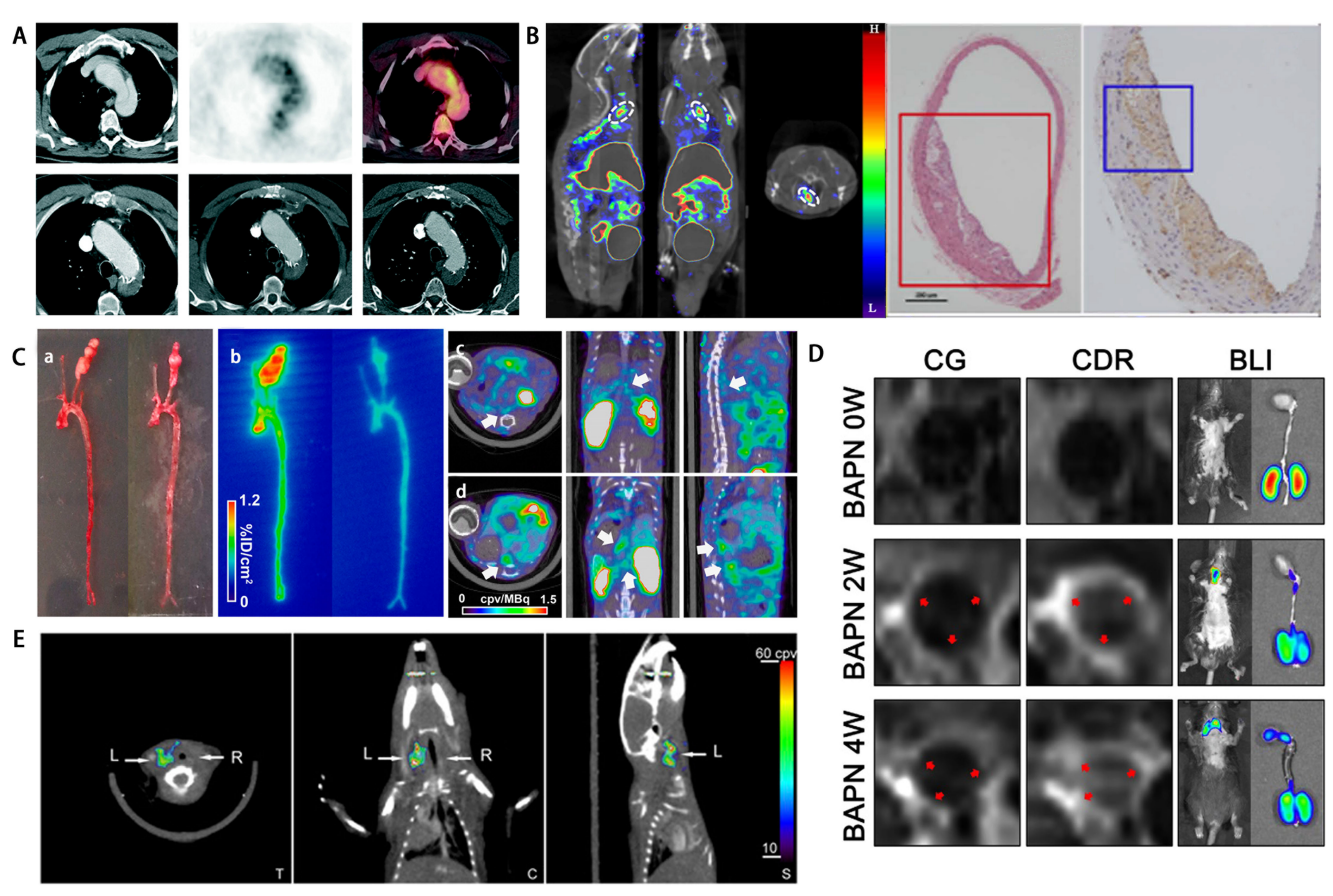
**Application of different molecular targets in different imaging techniques**. (A) The FDG-uptake in the dissected aortic wall shown in CT, PET, PET/CT and 2 months, 2 years, 3 years follow-up with CT. Reproduced with permission from BMJ Publishing Group Ltd. [43]. ${\mathrm{(B)}}^{99\,m}$Tc-MAG3-anti-CD11b SPECT/CT images with pathological and immunohistochemical confirmation. Reproduced under CC-BY 4.0 license from Springer Nature [56]. (C) ${\mathrm{(a,b)}}^{99\,m}$Tc-RYM1 imaging of carotid aneurysm, carotid arteries *ex vivo* photography (a) and autoradiography (b) without (left) and with (right) pre-injection of excess of MMP inhibitor RYM; ${\mathrm{(c,d)}}^{99\,m}$Tc-RYM1 SPECT/CT images of abdominal aortic aneurysm animals model, (c) low remodeling group, (d) aneurysm group, Arrows: areas of maximal tracer uptake in aorta. This research was originally published in *JNM*. Toczek J *et al*. [57] Preclinical Evaluation of RYM1, a Matrix Metalloproteinase-Targeted Tracer for Imaging Aneurysm. J Nucl Med. 2017; 58: 1318–1323. © SNMMI . (D) Representative MRI images of mice after injection with CG and CDR, following BAPN administration for 0, 2, and 4 weeks. Mice were examined by BL after MR imaging. The red arrows for the CG or CDR groups indicate the same position. CG: DOTA-Gd, CDR: Col-IV-DOTA-RhB, BLI: bioluminescence imaging. Reproduced under CC-BY 4.0 license from Ivyspring International Publisher [58]. (E) *In vivo* imaging of MMP activation in aneurysm. left (L): carotid arteries aneurysmal; right (R): control. This research was originally published in JNM. Razavian M *et al*. [59] Molecular imaging of matrix metalloproteinase activation to predict murine aneurysm expansion *in vivo*. J Nucl Med. 2010; 51: 1107–1115. © SNMMI .

Inflammatory responses accelerate metabolic processes by increasing the consumption of glucose. PET imaging with a radioactive tracer, 18-fluoro-2-deoxyglucose (FDG) can detect the high expression area, which has been shown to be correlated with the presence of macrophages and related to the risk of AD progression (Fig. 2A, Ref. [43, 56, 57, 58, 59]) [43, 60]. A study with PET/CT identified that translocator protein (TSPO) as a diagnostic technique in cardiovascular disease [61]. Also, TSPO expressions can be utilized to assess populations of macrophage during pathological progression [62]. Mac-1 is also a specific receptor of superparamagnetic iron oxide nanoparticles (SPIONs), which accumulate into macrophages by endocytosis and can be detected by MRI [63]. In another study, Mac-1 was used as the central biomarker in a mouse model of atherosclerosis, and a nuclear imaging ${\mathrm{probe,}}^{99\,m}$Tc-MAG3-anti-CD11b, was developed to assess inflammatory status with single photon emission computed tomography/computed tomography (SPECT/CT). Histological anatomy and section staining verified the formation of atherosclerotic plaques and Mac-1 high expression in the areas displayed by SPECT/CT (Fig. 2B) [56]. In our experiment, anti-CD11b-TCO/Tz-PEG11-HYNIC-99mTc, a pre-targeting imaging molecular probe has been established and which allowed the SPECT/CT image detection of CD11b infiltration in progressive AA [64] (Table 1, Ref. [43, 56, 57, 58, 59, 60, 61, 63, 65, 66]).

**Table 1.** **Summary of molecular targets in pathophysiology of AAs with Imaging Techniques**.

*Molecular targets*	*Imaging techniques*	*Materials*
*Inflammatory cells*	PET/CT	FDG [43, 60]
	PET/CT	TPSO [61]
	SPECT/CT	^99m^Tc-${\mathrm{MAG}}_{3}$-anti-CD11b [56]
	MRI	SPIONs [63]
*MMPs*	SPECT/CT	^99m^Tc-RP805 [59, 65]
	PET/CT	^99m^Tc-RYM1 [57]
*Apoptosis*	SPECT/CT	^99m^Tc-Duramycin [66]
*Col-IV*	MRI or fluorescence imaging	Col-IV-DOTA-Gd-RhB [58]
*miRNAs*	-	-

Inflammation may lead to increased C-reactive protein (CRP) levels. One study investigated the relationship between serum CRP-to-albumin ratio (CAR) and progression in patients with AAA, and the results indicated that increased serum CAR was significantly associated with larger diameter [67]. However, changes in inflammatory factors in blood are subject to a variety of diseases and lack specificity for AAs. The imaging technology combined with inflammatory markers is more intuitive and effective.

### 3.2 Matrix Metalloproteinases (MMPs)

MMPs are endopeptidases secreted by several matrix-resident and inflammatory cells of the vessel wall and belong to the metzincins superfamily, which contain a wide spectrum of zinc-dependent elastases and collagenases [68]. Specific endogenous inhibitors of MMPs, tissue inhibitors of metalloproteinases (TIMPs), and MMPs are indispensable for natural physiological ECM remodeling, but the dysregulated interaction between two endopeptidases results in ECM degradation [69, 70]. MMPs not only lead to ECM degradation but also act on non-matrix substrates [71]. Matthew and colleagues [72] have demonstrated that MMPs mediate proteolysis by regulating immune-inflammation responses, promoting the migration of inflammatory cells and modulating non-matrix molecules. Moreover, MMPs play a vital role in the recruitment of inflammatory cells, migration of VSMCs into the vessel intima and promotion of neovascularization [73].

Although MMPs are a spectrum of enzymes that have similar functions, each MMP has specific functions and is expressed at different levels in different tissues during inflammation [69]. Particularly, in patients with AAs, MMP-2/9/12 expression have been shown significantly higher in serum and aortic tissue, regardless of the genetic predisposition [74, 75, 76]. Distinct from other MMPs, MMP-12 is exclusively secreted by macrophages and could regulate the degradation of elastin and type IV collagen [77]. The enzyme can induce the activation of MMP-2 and MMP-3, which both activate MMP-7, MMP-8, and MMP-13. This amplifying cascade effect results in excessive degradation of ECM [78].

MMPs have three complexes of zinc ions in the catalytic center, and they are exposed only when MMPs are activated. Thus, the structural changes associated with MMP activation can be exploited for molecular diagnosis. In the early stage, antibody probes specific to the activation-specific epitopes had low specificity for MMPs. Importantly, each MMP has specific substrates. Therefore, substrate specificity can be exploited to enhance the selectivity to target MMPs [79]. Inhibitor-based probes are another technology to identify MMPs and obtain good results [80, 81]. Research with PET/CT reported an MMP inhibitor, RYM, and demonstrated that 99mTc-RYM1 probes are more abundant in AAA (Fig. 2C) [57]. In the SPECT technique, the probes were designed with special tracers of MMPs, such as RP782 and RP805. 99mTc-RP805 signal was significantly higher in the inflammatory area (Fig. 2E) [59, 65]. Serum MMPs levels, specifically MMP-2 and MMP-9, were shown to be associated with AAs. Studies on serum MMP levels are based on patients who have been clinically diagnosed, which may be significant for risk assessment, but inadequate to predict AAs [82, 83]. Thus, MMPs could be a possible biomarker to visualize inflammation by molecular imaging for early diagnosis and may serve as a potential target for treatment (Table 1).

### 3.3 Apoptosis

Recent evidence has shown that apoptosis is another vital process in the pathogenesis of AAs [84, 85]. VSMC is the main effector cell constituting the aorta tunica media and play an essential role in maintaining vascular wall structure and function [86]. Several studies have demonstrated that the inflammatory cells induce apoptosis and MMP synthesis [42]. Moreover, endothelial injury mediates the up-regulated expression of long non-coding RNA H19 and Toll like Receptors (TLRs). H19 mediates the expression levels of the transcription factor HIF1α, which has been shown to activate apoptosis of VSMCs. The TLR4-related signaling pathways increase the expression of the MMP-9 level, consequently stimulating the VSMCs dysfunction. VSMC apoptosis is considered to weaken the aortic structural integrity and participates in the pathogenesis of AAs [87, 88]. Additionally, SPECT with a 99mTc-duramycin probe was effective in evaluating apoptosis in AAs [66] (Table 1).

### 3.4 Type IV Collagen (Col IV)

Histological studies have shown that collagen is an important component of the ECM, which strengthens the aortic wall structure and affects cell proliferation and adhesion by binding to integrins [89]. Moreover, collagen regulates the local activity of cytokines [90]. Col IV is one of component of the subendothelial basement membrane of the tunica intima in the aortic wall. It has been demonstrated that medial degeneration leads to long-term chronic stimulation, which results in intimal tearing of AD [8, 91]. Studies have also shown that Col IV is exposed initially at sites of endothelial injury [92]. Meanwhile, the activation of MMP-2/9 degrades basement membrane proteins expression in SMCs, including Col IV [75, 93].

Col IV belongs to the subendothelial basement membrane and is exposed on the aortic intima of early phase AAs. Recent research has demonstrated the MRI and fluorescence imaging with a Col IV-targeted probe, Col-IV-DOTA-Gd-RhB, to visualize aortic lesions, which can effectively predict AD and rupture of AAs (Fig. 2D) [58] (Table 1).

### 3.5 Others

As the first biomarker identified in 1993, microRNAs (miRNAs) had been reported to contribute to both physiological and pathologic processes of cardiovascular diseases [94]. Overexpression of miRNAs, such as miR-29, -195, -21 and -143/145. miR-29 and miR-195 lead to the degradation of ECM and decrease miR-21 [95]. MiR-21 is associated with transforming growth factor β (TGF-β) signaling pathway dysfunction [96, 97]. MiR-1 43/145 promotes a phenotypic switch of VSMC and induces degeneration of the medial layer [98]. It has been demonstrated that miRNAs are strikingly stable in human plasma/serum [99]. In patients with AAs and AD, miRNAs show over 10- to 40-fold increases in plasma [100].

Additionally, TGF-β signaling molecules and TGF-β receptors (TGFBR) play a multitude of roles in various physiological processes. TGF-β signals activate Smad2/3 through TGFBR to induce the phosphorylation of Smad2/3 proteins. Moreover, TGF-β signals can be mediated by non-Smad pathways, which can transduce signals from Ang II and be mediated by mitogen-activated protein kinases [101]. Furthermore, the TGF-β and Ang II pathways can modulate vascular tone and the SMC phenotype by interacting with each other [9]. Mutations in the TGF-β pathway are associated with Marfan syndrome and Loeys–Dietz syndrome [102].

## 4. Molecular Targets to Treat AA

### 4.1 Medicine

#### 4.1.1 Traditional Medicine

The current medicinal managements of AAs, mainly focus on controlling blood pressure and heart rate as an adjuvant therapy. ꞵ-Adrenergic blockade (β-blockers) has been used as a medicinal treatment in patients with AAs for decades and the effectiveness of β-blockers in reducing aortic aneurysm growth rate in turkeys was demonstrated over 70 years ago [6]. Moreover, β-blockers showed a positive result reducing the rate of change in central aortic pressure [103]. Several randomized clinical trials have shown that in patients with Marfan syndrome, β-blockers decrease the dilation of the aortic root and aortic complications [104]. According to the guidelines, all patients with AAs and Marfan syndrome should be administered β-blockers unless contraindicated [5, 6]. Heart rate is the major determinant of the dosage of β-blockers. Despite limited evidence for its specific target in AAs pathology, β-blocker therapy is still widely used as a first-line medicine to prevent the progression of aortic dilation. Several large randomized clinical trials in patients with Marfan syndrome have subsequently found that the evidence is inadequate to suggest that losartan is better than β-blocker therapy in reducing aortic dilation. However, this therapy is still controversial because of the differences in results of mouse models and human studies [105, 106].

#### 4.1.2 Inhibition of Inflammation

**Fig. 3.**
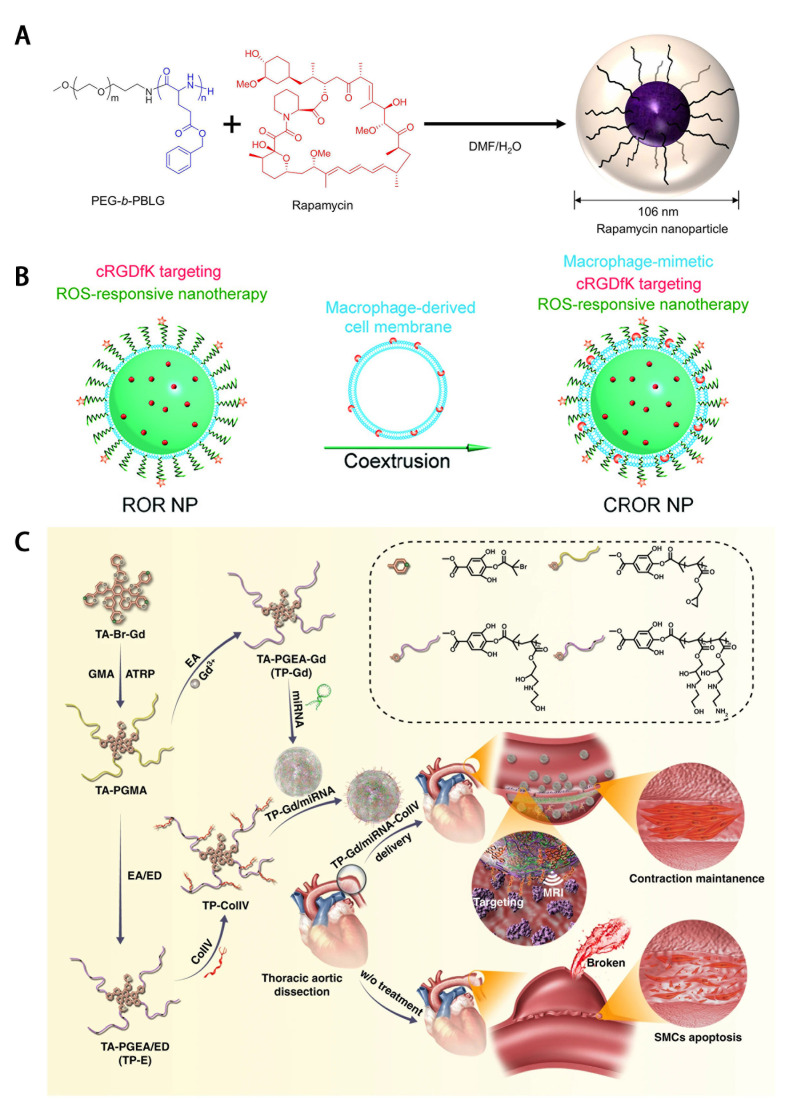
**Nanoparticles of different molecular targets**. (A) Structure of rapamycin-incorporated nanoparticles. Reproduced under CC-BY 4.0 license from Plos One [107]. (B) Schematic of ROS-responsive nanoplatform. Reproduced with permission from Elsevier [108]. (C) Schematic diagram illustrating the preparation of TP-Gd/miRNA-ColIV complexes and the resultant targeted gene therapy. Reproduced under CC-BY 4.0 license from Springer Nature [109].

Rapamycin had been used as a potent anti-inflammatory drug in the clinic. Rapamycin- were used for occlusive cardiovascular diseases with coated balloons and stents. One group designed nanoparticles (NPs), PEG-b-PBLG NPs, for targeted rapamycin delivery. In animal experiments, macrophages preferentially ingested NP. This NP delivery system effectively led to mitigation of aortic pathological process under a low dosage of rapamycin. Furthermore, the rapamycin-loaded PEG-b-PBLG NPs significantly reduced inflammatory cytokine production and MMP activity (Fig. 3A, Ref. [107, 108, 109]) [107]. On the other hand, another group fabricated a ROS-responsive NP to deliver rapamycin. These NPs acted as a ROS scavengers to inhibit oxidative stress and apoptosis of VSMC in the *in-vitro* experiments (Fig. 3B) [110]. Additionally, the rapamycin-loaded formulation reduced macrophage recruitment, MMP activity and ECM deterioration [108].

#### 4.1.3 Inhibition of MMP

Several types of MMPs are expressed in the pathological aortic tissue of AAs. Synthetic MMP inhibitors are specific molecularly targeted drugs to decrease MMP activity and prevent the pathological changes of aortic diseases [110]. Studies have shown that doxycycline reduced MMP activity by decreasing the mRNA stability of MMP-2 [111]. Many preclinical studies have demonstrated the potential application of doxycycline to impede the development of aneurysms [112].

Hydroxamate based medicines such as marimastat, prinomastat and batimastat (BB-94) are another group of synthetic MMP inhibitors, which bind zinc atoms [113]. BB-94was tested clinically to reduce MMPs in advanced malignancy as the first synthetic MMP inhibitors [114]. One study utilized NP delivery systems, EL-PEG-PLA NPs, to target BB-94 in a rat aortic injury mode. The results indicated that BB-94 greatly improved efficacies of reducing elastin degradation, calcification, and aortic dilation [115].

#### 4.1.4 Protection of the Elastin Matrix 

Clinical evidence supports that the TGF-β signal is vital in AD formation, although the results are largely based on end-stage diseased aortae [116]. TGF-β1 shows multiple functions in regulating processes of cell growth. TGF-β1 is an established elastogenic factor at a low concentration [117]. On the other hand, a higher concentration of TGF-β1 may promote calcification by differentiating of SMC to osteogenic phenotype. This group developed PLGA NPs to deliver TGF-β1 and doxycycline. In an *in vitro* study, VSMCs were cultured in a 3D matrix and loaded with NPs, and the results showed that the NPs remarkably increase the elastin content and matrix assembly [118].

An previous investigation showed that oligomers of hyaluronan (HA-o) improved elastin assembly [119]. However, HA-o is susceptible to proteolysis in the hyperactive of MMP environment and has a short blood half-life time [120]. Onoda and colleagues designed a HA-o- loaded PLGA NP to avoid these deficiencies. The HA-o-NPs shown positive results that it could provide continuous and controlled payload release. In an *in vitro* experiment, VSMCs showed increased elastin matrix deposition and lysyl oxidase activity after treatment with HA-o-NP [121].

#### 4.1.5 Others

Nucleic acids are indispensable for functional cells; thus, nucleic acid dysregulations contribute to the initiation of different diseases. As previously mentioned, miRNAs have been suggested as biomarkers for the diagnosis and treatment of AAs [122]. For example, miR-145 is an upstream factor in the regulation of KLF4, which is a vital transcription factor associated with the phenotypic switching of VSMCs responsible for the degeneration of the medial layer [98, 123]. Increased miR-145 reduces KLF4 expression, maintains VSMC stability in a contractile phenotype, and prevents enlargement of the aorta [124]. MiR-126 is another factor that regulates vascular cell adhesion molecule-1 (VCAM-1) expression [125]. VCAM-1 is related to the migration of inflammatory cells to inflamed ECs as an endothelial adhesion molecule upregulated in AAs [126]. Upregulation of miR-126 contributes to modulating aortic wall inflammation and integrity [127, 128].

### 4.2 Delivery System

Although molecularly targeted medicines show specificity and effectiveness against specific target molecules, systemic delivery may activate or inhibit the molecules that are essential for normal homeostasis. Meanwhile, the effectiveness of molecularly targeted medicines is limited because of its poorly water-soluble and requirement for parenteral administration, such as rapamycin and MMP inhibitors [129, 130]. Therefore, an appropriate delivery system is required to reduce side effects of molecularly targeted drugs while maintaining the efficacies.

The ever-increasing use of NPs in biomedicine reflects the great advances in novel imaging and drug delivery systems. In addition, NPs can be easily functionalized and applied in a variety of diseases [131]. Exosomes are the smallest membrane-delimited extracellular vesicles released by cells [132]. Exosome mainly function as regulators of cell-to-cell communication at short or long distances [133]. It has been used as a biomarker to monitor the progression of diseases [132, 134]. Exosomes have unique endogenous features and biological properties, such as stability in body fluids, immune tolerance and the ability to carry RNAs, DNAs, and proteins naturally [135, 136]. In addition, exosomes range from 50 to 150 nm in size, which can escape macrophage phagocytosis and promote permeation across biological barriers [137]. Because of these advantages, exosomes can be a suitable candidate for drug delivery as NPs. However, the low production quantity and difficulty of isolation, drug loading and delivery efficiency limit the application of exosomes in clinical practice [138, 139]. To address this problem, several studies have focused on synthetic NPs, which are easier to prepare and more efficient in drug encapsulation and delivery (up to nearly 90%) [139, 140]. However, synthetic NPs may induce a stronger immune response than exosomes [131].

The delivery system based on NPs is primarily used for molecularly targeted drugs, targeted antibodies or peptides and may be coated with molecules to protect the potency of the drugs or to enhance their effect [108, 129]. A study reported a TP-Gd/miRNA-Col IV NP delivery system, which is a multifunctional nucleic acid delivery nanosystem [109]. This nanosystem can be visualized by MRI and deliver nucleic acid and targeted peptide Col IV simultaneously (Fig. 3C). In addition, this nanosystem can efficiently deliver miR-145 to stabilize the vascular structure. In the paper, nanosystem was successfully prepared and used for predicting and monitoring AD [109]. As previously mentioned, the NPs loaded with BB-94 successfully released BB-94 in the lesion region, which inhibited local MMP activity and suppressed aortic dilation at small doses [115]. Poly (lactic-co-glycolic acid) has been used in nanotechnology applications, such as targeted and controlled drug delivery [141]. As an application of poly (lactic-co-glycolic acid), a doxycycline-loaded poly(lactic-co-glycolic acid) NP delivery system has been developed. It has been demonstrated that elastin have the potential to bind and inhibit MMPs to prevent the progression of AAs [112].

## 5. Conclusions

The current management of AAs has drastically reduced morbidity and mortality. The current treatment decision for AAs is primarily based on the clinical symptoms and physical factors of the aorta. However, these indicators are insufficient for predicting outcome and risk stratification. Surgical repair is the first option for AAs. However, studies on the pathophysiological basis of AAs have found that numerous molecules might be involved in its pathogenesis, and molecular probes have the potential to solve this vexing problem. In our review, we summarized two highlights from the development of molecular probes. Firstly, the probes integrated molecular targets for diagnosis and risk assessment in the subclinical stage. Secondly, molecular probes with NPs can be adapted to accommodate the complexity of AAs pathogenesis mechanisms and manifestations, achieve drug intervention and may accomplish precision medicine. As research on molecular targets further develops, it is conceivable that early diagnosis and personalized treatments at the molecular level may become a reality.

## Abbreviations

AA, aortic aneurysm; TAA, thoracic aortic aneurysms; AAA, abdominal aortic aneurysm; AD, aortic dissection; CT, computed tomography; MRI, magnetic resonance imaging; ECM, extracellular matrix; VSMC, vascular smooth muscle cell; BAV, bicuspid aortic valve; WSS, wall shear stress; CMR, cardiovascular magnetic resonance; TAV, tricuspid aortic valve; PET, positron emission tomography; FDG, 18-fluoro-2-deoxyglucose; TSPO, translocator protein; SPION, superparamagnetic iron oxide nanoparticle; SPECT, single photon emission computed tomography; CRP, C-reactive protein; CAR, CRP-to-albumin ratio; MMP, matrix metalloproteinase; TIMP, tissue inhibitors of metalloproteinase; TLRs, Toll like Receptors; Col IV, type IV collagen; TGF-β, transforming growth factor β; NP, nanoparticle; BB-94, batimastat;HA-o, oligomers of hyaluronan; VCAM-1, vascular cell adhesion molecule-1.
